# Fat Body Organ Culture System in *Aedes Aegypti*, a Vector of Zika Virus

**DOI:** 10.3791/55508

**Published:** 2017-08-19

**Authors:** Hae-Na Chung, Stacy D. Rodriguez, Victoria K. Carpenter, Julia Vulcan, C. Donovan Bailey, Madhugiri Nageswara-Rao, Yiyi Li, Geoffrey M. Attardo, Immo A. Hansen

**Affiliations:** ^1^Department of Biology, New Mexico State University; ^2^Department of Molecular Genetics and Microbiology, Duke University; ^3^Department of Computer Sciences, New Mexico State University; ^4^Department of Epidemiology of Microbial Diseases, Yale School of Public Health; ^5^Institute of Applied Biosciences, New Mexico State University

**Keywords:** Infectious Diseases, Issue 126, Fat body, organ culture, mosquitoes, *Aedes aegypti*, nutrition, vitellogenesis

## Abstract

The insect fat body plays a central role in insect metabolism and nutrient storage, mirroring functions of the liver and fat tissue in vertebrates. Insect fat body tissue is usually distributed throughout the insect body. However, it is often concentrated in the abdomen and attached to the abdominal body wall.

The mosquito fat body is the sole source of yolk proteins, which are critical for egg production. Therefore, the *in vitro* culture of mosquito fat body tissues represents an important system for the study of mosquito physiology, metabolism, and, ultimately, egg production. The fat body culture process begins with the preparation of solutions and reagents, including amino acid stock solutions, *Aedes* physiological saline salt stock solution (APS), calcium stock solution, and fat body culture medium. The process continues with fat body dissection, followed by an experimental treatment. After treatment, a variety of different analyses can be performed, including RNA sequencing (RNA-Seq), qPCR, Western blots, proteomics, and metabolomics.

In our example experiment, we demonstrate the protocol through the excision and culture of fat bodies from the yellow fever mosquito, *Aedes aegypti*, a principal vector of arboviruses including dengue, chikungunya, and Zika. RNA from fat bodies cultured under a physiological condition known to upregulate yolk proteins versus the control were subject to RNA-Seq analysis to demonstrate the potential utility of this procedure for investigations of gene expression.

**Figure:**
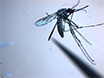


## Introduction

Mosquitoes are vectors of devastating human diseases, including malaria, dengue fever, chikungunya, and Zika^1^,^2^,^3^. Despite intense international efforts to curb these diseases and to control disease-transmitting mosquito populations, epidemic outbreaks of mosquito-borne diseases are still common, especially in developing countries. Effective vaccines against many of these diseases are either unavailable or of limited efficacy^4^,^5^. The most effective way to prevent outbreaks is to control mosquito populations, mainly through the use of insecticide treatments. However, insecticide resistance has developed in many mosquito populations and become a common problem around the world^6^,^7^,^8^. The study of mosquito physiology is essential to the development of novel tools and strategies to control disease.

The mosquito fat body plays a central role in nutrient storage, metabolic homeostasis, reproduction, and xenobiotic catabolism^9^,^10^,^11^,^12^. It is the main storage organ for triglycerides, glycogen, and amino acids in form of storage proteins. It also functions as the location of synthesis for most hemolymph proteins and metabolites. In mosquitoes, the fat body is the sole source of yolk protein production that occurs in females after they take a blood meal^13^,^14^.

The principal cell type of the fat body is the large, polyploid trophocyte or adipocyte^3^,^9^,^10^,^12^. Fat body tissue is organized into lobes or sheaths and can be found in all body parts of the mosquito, with the largest portion located in the abdomen, where large lobes of fat body are attached to the abdominal body wall.

The mosquito fat body culture system presented here was developed in the '70s and remains a powerful tool for studying fat body physiology^10^, especially in combination with current analysis technologies. The foundation of this technique is based on the isolation of the abdominal body walls and the associated fat body tissue. The hydrophobic nature of the abdominal cuticle causes it to float on the surface of the culture medium, with the attached lobes of abdominal fat body immersed. The spiracles and tracheolar structure are maintained, ensuring oxygenation of the cultured tissue. Henceforth, we will refer to these preparations as "fat bodies." Isolated fat bodies remain viable for more than 12 h when incubated in the appropriate medium (unpublished results). Fat body culture is a valuable tool that has addressed a variety of questions regarding fat body endocrinology and physiology^9^,^10^,^12^,^15^,^16^,^17^.

Cultured fat bodies can be subjected to various experimental and control treatments, the timing of which can be decided upon by the investigator. At the end of the incubation period, the fat bodies can be collected and processed for downstream analyses, including qPCR^16^,^17^,^18^,^19^, Western blotting^18^,^20^, proteomics^21^, or metabolomics^22^. Experiments can be performed on different scales, from individual fat bodies to groups of hundreds that can be cultured together.

The representative results included here were derived from fat bodies cultured in the presence of amino acids and the steroid hormone 20-hydroxy ecdysone to simulate the blood meal activation of vitellogenesis^16^,^17^,^23^,^24^. We analyzed and compared the differential gene expression of not-activated versus activated fat bodies via next-generation sequencing analysis.

## Protocol

### 1. Preparing the Solutions and Reagents

1. **Amino acid stock solution**
  1. Prepare a 4X amino acid stock solution^23^,^24^ by weighing and adding the 20 different amino acids to an Erlenmeyer flask according to the concentrations given in **Table 1**. NOTE: In some cases, amino acid(s) can be excluded from the solution and replaced by equal molar amounts of mannitol to maintain an equal concentration of osmolytes.
  2. Add the appropriate amount of ddH_2_O to produce the desired volume of solution.
  3. To ensure that the amino acids have completely dissolved, heat gently while continually stirring until the liquid is completely clear; it will take on a slight yellow hue. NOTE: It may be necessary to reduce the pH to 6 by adding HCl to allow some of the more hydrophobic amino acids to dissolve.
  4. Aliquot the solution and sterile-filter using a syringe filter with a 0.2-µm pore size.
  5. Store in a sealed container at -20 °C for up to 6 months.
2. **APS** Note: See^25^.
  1. For the 20X salt stock solution, combine the salts listed in **Table 2** in 50 mL of water. Stir until completely dissolved. NOTE: It may be necessary to slightly heat the solution to completely dissolve the salts.
  2. Sterile-filter using a syringe filter with a 0.2-µm pore size and store the solution in a 50-mL tube at -20 °C.
3. **Calcium stock solution**
  1. For the 50X calcium stock solution, add 0.90 g of calcium chloride to 100 mL of water (**Table 3**); stir until completely dissolved.
  2. Sterile-filter using a syringe filter with a 0.2-µm pore size, aliquot the solution into 50-mL tubes, and store at -20 °C.
4. **Tris buffer (pH 7.4)**
  1. For the Tris buffer, combine the salt and solutions listed in **Table 4** and add ddH_2_O up to 100 mL.
  2. Sterile-filter using a syringe filter with a 0.2-µm pore size and aliquot the solution into 50-mL tubes; store at room temperature.
5. **Fat body culture medium**
  1. To prepare the fat body culture medium, prepare all solutions listed above.
  2. Combine them as per the volumes in **Table 5** to make 200 mL of fat body culture medium.
  3. Adjust the pH to 7.2 using NaOH or HCl.
  4. Sterile-filter the solution using a syringe filter with a 0.2-µm pore size.
  5. Divide the solution into 15-mL aliquots and store at -20 °C for up to 6 months.

### 2. Preparing for Dissection

1. Prepare a stereomicroscope (10-20x magnification) with illumination and lay out two ultra-fine tweezers and a pair of micro dissection scissors.
2. Prepare all solutions necessary for the experiment. Thaw the fat body culture medium to room temperature. NOTE: Precipitants can be dissolved by gently heating the solution to no higher than 30 °C.
3. With an aspirator, collect adult female mosquitoes (3-7 days after emergence) and anesthetize them using carbon dioxide or ice. NOTE: Once anesthetized, the mosquitoes should be kept under CO_2_ for the shortest time possible, not exceeding 20 min. Alternatively, the mosquitoes can be anesthetized on ice and kept for approximately 30 min.
4. Prepare a 6-well plate with 3 mL of APS per well.

### 3. Fat Body Dissection

1. Focus the dissecting stereomicroscope and adjust the chair to a comfortable height.
2. Place a concave microscope slide on the microscope surface and add two drops of APS to the center.
3. Pick up a mosquito by a leg using a forceps and transfer it to the APS surface on the microscope slide.
4. Carefully grip the mosquito by the thorax, with the forceps in the left hand, and rotate the mosquito so that the ventral side is upwards.
5. While holding the body steady by the thorax, grasp the last two abdominal segments, with the forceps in the right hand, and gently pull. NOTE: The last two abdominal segments will separate from the abdomen and the attached ovaries, Malpighian tubules, hindgut, and midgut; sometimes the crop will slide out of the abdomen. If they have not been removed, insert the closed tips of the forceps into the small hole created and remove the remaining tissues.
6. Set the right forceps aside and pick up the spring scissors. Slide one blade into the hole in the abdomen up to the segment below the thorax. Gently and swiftly cut the abdomen lengthwise. NOTE: Once the cut has been made, the abdomen should begin to expand outward, though this may not occur immediately.
7. Proceed to make the second cut, which will be a lateral cut below the thorax and slightly below where the first cut ended. NOTE: The abdomen should then open up and dissociate from the thorax, with the cuticle up and the fat bodies side-immersed within the APS. This abdominal body wall is the fat body preparation for *in vitro* culture.
8. Lift the fat bodies from the APS solution using the tip of a pair of forceps and transfer them from the solution to the 6-well plate containing APS at room temperature. Allow the tissue to rest for approximately 0.5 h before advancing to the fat body culture.

### 4. Fat Body Culture

1. Incubate the fat bodies on APS at room temperature for at least 0.5 h to equilibrate them.
2. Transfer the fat bodies using the tip of the forceps and gently lift them out of the APS solution. Lower the tip into the fat body culture medium. NOTE: The fat body should open on the surface of the medium and float freely. The fat body culture is typically performed in 96-well plates, with 150-200 µL of medium in each well. One well can fit up to three individual fat bodies, and they are viable for more than 12 h (unpublished personal results).
3. After the incubation period, transfer the fat bodies from the medium into 1.5-mL centrifuge tubes containing the appropriate reagents for downstream processing. NOTE: Typical analyses include qPCR^16^,^17^,^18^,^19^, Western blotting^18^,^20^, transcriptomics^26^, proteomics^21^, or metabolomics^22^.

## Representative Results

As an example, we performed a fat body culture experiment and stimulated isolated fat bodies by incubating them on a solution containing a balanced mixture of all twenty naturally occurring amino acids and the insect steroid hormone 20-hydroxy ecdysone (10 µM) for 6 h. As a control, fat bodies were incubated on APS for an equal amount of time.

After incubation, the total RNA was isolated using a tri-reagent^27^ following the manufacturer's instructions. The quality and quantity of extracted RNA samples were assessed using a spectrophotometer, fluorometric quantitation, and agarose gel electrophoresis. RNA sequencing libraries were generated using 4 µg of total RNA and were quantified using two different techniques. Subsequently, the libraries were sent to a commercial provider for paired end-sequencing.

The results of this experiment are shown in **Table 6**. Genes showing the strongest transcriptional response to amino acid and 20-hydroxyecdysone were primarily yolk protein genes, which is in agreement with previous results^11^.

Figure 1 shows a heat map indicating the gene expression levels of 1,256 differentially expressed genes from cultured fat bodies after two different treatments.

**Figure:**
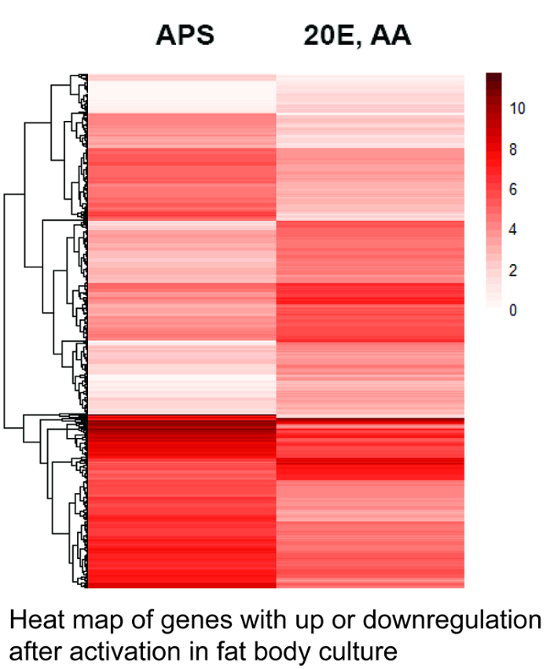

Figure 1**. Heat map of genes expressed in the fat body culture.** The heat map was calculated based on the number of specific transcripts for each gene in the different libraries using the heatmap package^28^ that is part of the R software environment. The darker shade represents higher gene expression. 1,256 genes with statistically significant variation in expression (Q-values < 0.05) are shown. The genes are ordered according to their averaged expression levels, indicated by the dendrogram on the left (not according to their phylogenetic relationships). Note the high number of genes with up- or down-regulated expression after stimulation with amino acids (AA) and 20-hydroxyecdysone (20E). APS = *Aedes* physiological saline. See **Supplemental File 1 **for a list of genes and their relative expression levels. Please click here to view a larger version of this figure.

**Table:** 

**Amino Acid**	**Molecular Weight g/mol**	**mM Concentration**	**mg per Liter**	**mg for 300 mL volume**
**Alanine**	89.1	26.68	2377.19	713.16
**Arginine**	174.2	26.68	4647.66	1394.3
**Asparagine**	150.1	26.68	4004.67	1201.4
**Aspartic Acid**	133	26.68	3548.44	1064.53
**Cysteine**	121.16	10.68	1293.99	388.2
**Glutamic Acid**	147.1	26.68	3924.63	1177.39
**Glutamine**	146	26.68	3895.28	1168.58
**Glycine**	75	53.32	3999	1199.7
**Histidine**	155.16	80	12412.8	3723.84
**Isoleucine**	131	10.68	1399.08	419.72
**Lycine**	183	26.68	4882.44	1464.73
**Leucine**	131	26.68	3495.08	1048.52
**Phenylalanine**	165	10.68	1762.2	528.66
**Proline**	115	26.68	3068.2	920.46
**Serine**	105	53.32	5598.6	1679.58
**Threonine**	119	10.68	1270.92	381.28
**Tryptophan**	204	10.68	2178.72	653.62
**Tyrosine**	181	5.32	962.92	288.88
**Valine**	117	10.68	1249.56	374.87
**Methionine**	149	10.68	1591.32	477.4

**Table 1. 4X Amino acid stock solution.**

**Table:** 

**Component**	**Weight in grams added to 50 ml ddH_2_O**
**NaCl**	8.0 g
**KCl**	0.074 g
**MgCl_2_-6H_2_O**	0.120 g
**NaHCO_3_**	0.0250 g

**Table 2.** **20X salt stock solution.**

**Table:** 

**Component**	**Weight in grams added to 100 ml ddH2O**
**CaCl_2_-2H_2_O**	0.90 g

**Table 3.** **50X calcium stock solution.**

**Table:** 

**Component**	**Concentration of stock solution**	**Volume stock for 100 ml buffer**
**Tris pH8.0 **	1 M	5 mL
**EDTA**	0.25 M	2 mL
**NaCl**	NA	0.3 g
**ddH_2_O**	NA	to 100 mL (~93 mL)

**Table 4.** **Tris buffer.**

**Table:** 

**Component**	**Volume stock for 200 ml **
**Amino Acid Stock Solution**	150 mL
**Salt Stock Solution**	10 mL
**Calcium Stock Solution**	4 mL
**TES Buffer**	10 mL
**ddH2O**	26 mL

**Table 5. Fat body culture medium**.

**Table:** 

**Annotation**	**Gene description**	**Fold change**	**P-value**
AAEL006138	*Vitellogenin-B*	3443	2.52E-112
AAEL006126	*Vitellogenin-C*	2795	8.64E-91
AAEL006563	*vitellogenic carboxypeptidase*	1002	2.17E-119
AAEL010434	*Vitellogenin-A*	220	1.14E-27
AAEL006542	*vitellogenic carboxypeptidase*	185	2.14E-65
AAEL012678	*AAEL003006-PA [Aedes aegypti](65%)*	96	4.00E-70
AAEL000080	*hypothetical protein *	82	6.69E-188
AAEL015312	*Vitellogenic cathepsin B*	77	1.27E-15
AAEL009588	*nuclear receptor 3*	75	4.58E-56
AAEL010529	*hypothetical protein*	66	1.32E-29

**Table 6.** **Experimental results.**

**Supplemental File 1.** Please click here to download this file.

## Discussion

Insect organ culture was used extensively to study insect endocrinology, development, and metabolism, as well as to investigate the interaction between specific organs and bacterial symbionts^29^,^30^,^31^,^32^,^33^,^34^. *In vitro* fat body organ culture was used specifically to study amino acid transport and the regulation of yolk protein production in mosquitoes and other *Diptera*^16^,^17^,^35^,^36^. During the process of vitellogenesis, the mosquito fat body uses an array of high-specificity amino acid transporters to import blood meal-derived amino acids from the hemolymph to synthesize large quantities of yolk proteins^12^,^19^,^35^,^36^. Fat body culture was instrumental to the delineation of the fat body nutritional requirements in this context^18^.

The quality of the starting material, female mosquitoes, is critical for the success of these experiments. Mosquito larvae raised in under-crowded conditions and fed on high-nutrient diets usually produce the best results. There are some important variables to consider when establishing mosquito fat body culture conditions in the laboratory in terms of experimental design. We showed in previous studies that fat body gene expression varies significantly depending on the individual life history and nutritional status of the mosquito^11^,^22^. The mosquito culture conditions should be uniform to reduce the variability in the size and nutritional reserves of the experimental mosquitoes. In addition, personnel performing the dissections should be trained to ensure rapid and accurate dissections with consistent results. Cell viability in isolated fat bodies can be checked using different staining methods^37^,^38^.

The experimental design of a fat body culture experiment should take into consideration the number of dissections possible in a given time period. When large quantities of fat bodies are required, multiple dissection sessions or multiple dissectors may be necessary. There is a wide range of future applications for *in vitro* fat body culture in mosquitoes and other insects. It will be especially useful for testing potential drug candidates for insect control. The use of transgenic techniques in insects to express specific reporter proteins in fat body trophocytes will open up new methods to develop powerful bioassays for the study of fat body physiology.

## Disclosures

We have nothing to disclose.
